# Updated Analysis of Complication Rates Associated With Invasive Diagnostic Procedures After Lung Cancer Screening

**DOI:** 10.1001/jamanetworkopen.2020.29874

**Published:** 2020-12-16

**Authors:** Hui Zhao, Ying Xu, Jinhai Huo, A. Cole Burks, David E. Ost, Ya-Chen Tina Shih

**Affiliations:** 1 Section of Cancer Economics and Policy, Department of Health Services Research, The University of Texas MD Anderson Cancer Center, Houston; 2 Department of Health Services Research, Management and Policy, College of Public Health Professions, University of Florida, Gainesville; 3 Now with WW HEOR-US Markets, Bristol Myers Squibb, Lawrenceville, New Jersey; 4 Department of Pulmonary and Critical Care Medicine, University of North Carolina at Chapel Hill, Chapel Hill; 5 Department of Pulmonary Medicine, The University of Texas MD Anderson Cancer Center, Houston

## Abstract

This case-control study compares complication rates associated with invasive diagnostic procedures before and after Medicare and Medicaid coverage of lung cancer screening tests.

## Introduction

On the basis of results from the National Lung Cancer Screening Trial (NLST),^1^ the Centers for Medicare and Medicaid Services began coverage of lung cancer screening with low-dose computed tomography (LDCT) in February 2015 for beneficiaries aged 55 to 77 years who meet screening eligibility criteria.^2^ A previous analysis found that complication rates of invasive diagnostic procedures for lung abnormalities in real-world settings were more than double those reported in the NLST.^3^ A major criticism of the previous analysis was that these estimates may not be directly applicable to the screening cohort because the study used claims data predating insurance coverage of LDCT screening. In this updated analysis, we evaluated the complication rates among patients who received lung cancer screening with LDCT in community settings.

## Methods

In this case-control study, we used *Current Procedural Terminology* (*CPT*) codes G0297 and S8032 from the IBM MarketScan Research Database (databases used: Commercial Claims and Encounters and Medicare Supplemental and Coordination of Benefits; IBM Corp) to identify patients who had LDCT screening between February 1, 2015, and June 30, 2017, and underwent an invasive diagnostic procedure within 6 months of LDCT. All patients in the study were required to be enrolled in private health insurance (including supplemental insurance for Medicare) 6 months before and after LDCT to calculate comorbidity scores and monitor the use of invasive procedures, respectively. We categorized invasive diagnostic procedures into the following groups: cytology or needle biopsy, bronchoscopy, thoracic surgery, and other surgical procedures (eTable 1 in the Supplement).^3^ We used* International Classification of Diseases, Ninth and Tenth Revision* diagnostic codes; *Current Procedural Terminology* code(s); or both (eTable 2 in the Supplement) to identify complications that occurred within 3 months of an invasive procedure and classified complications by severity (minor, intermediate, and major). We applied a 1:1 case-control direct matching method to generate our study cohort, with age, sex, comorbidity score, state of residence, and quarter-year of LDCT screening as matching factors. We followed the Strengthening the Reporting of Observational Studies in Epidemiology (STROBE) reporting guideline. As in the previous study,^3^ we report incremental complication rates, calculated as the difference in the rate between the case and control groups. This study was deemed exempt from the need for approval or patient informed consent by the institutional review board at MD Anderson Cancer Center because the MarketScan Research Database uses deidentified data.

## Results

**Figure 1.**
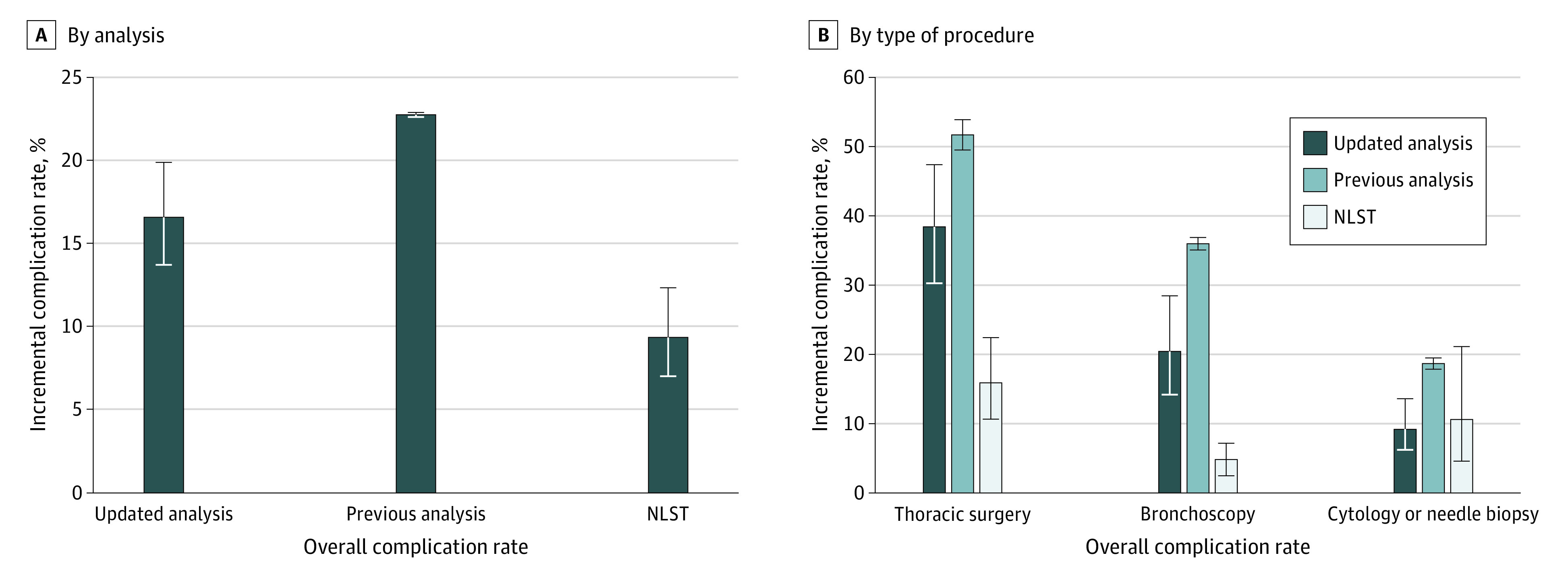
Comparison of Overall Complication Rates Estimated From the Updated Analysis, Previous Analysis, and National Lung Cancer Screening Trial (NLST) and by Type of Invasive Procedure A, The bars show the overall incremental complication rate at 3 separate times: the updated analysis (between February 1, 2015, and June 30, 2017); the previous analysis (patients who underwent diagnostic procedures between 2008 and 2013); and those included in the NLST (August 2002 through December 2009). B, Comparison of incremental complication rates by type of invasive procedure. The whiskers indicate 95% CIs.

**Figure 2.**
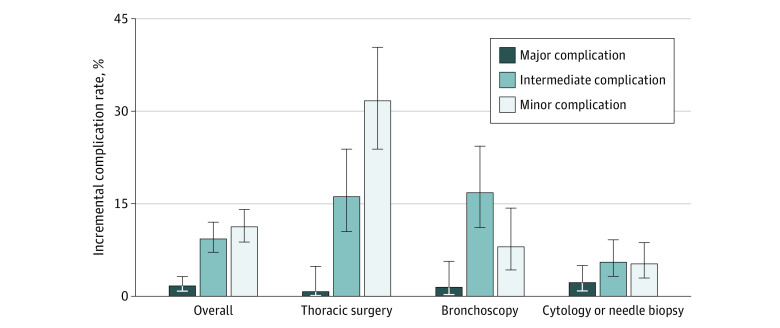
Incremental Complication Rate by Severity and Type of Invasive Diagnostic Procedure Data shown are for the updated analysis (between February 1, 2015, and June 30, 2017). The complication rate was based on all 4 types of invasive diagnostic procedures (cytology or needle biopsy, bronchoscopy, thoracic surgery, and overall). The whiskers indicate 95% CIs.

Among 18 887 patients (median age, 61 years [interquartile range, 58-63 years]; 9978 [52.8%] men) who had LDCT screening, 665 patients (3.5%) underwent invasive diagnostic procedures within 6 months after LDCT screening. We included 591 matched pairs in our case-control study. The overall incremental complication rate for all 4 types of invasive diagnostic procedures was 16.6% (95% CI, 13.7%-19.9%), which was less than the 22.8% (95% CI, 22.6%-22.9%) rate reported in the previous analysis but greater than the 9.4% (95% CI, 7.0%-12.3%) rate reported in the NLST (Figure 1A). Similar patterns were observed by procedure type. The incremental complication rates from our updated analysis were 38.5% (95% CI, 30.2%-47.4%) for thoracic surgery, 20.4% (95% CI, 14.2%-28.4%) for bronchoscopy, and 9.2% (95% CI, 6.2%-13.5%) for cytology or needle biopsy (Figure 1B). The overall complication rates by severity were 1.7% (95% CI, 0.9%-3.2%) for major, 9.3% (95% CI, 7.2%-12.0%) for intermediate, and 11.2% (95% CI, 8.8%-14.1%) for minor complications (Figure 2).

## Discussion

Screening with LDCT reduces lung cancer mortality in high-risk populations.^1^ The findings of this case-control study indicated an overall complication rate of 16.6% among patients who underwent invasive diagnostic procedures after lung cancer screening with LDCT in real-world circumstances. Most complications were of minor or intermediate severity. Several reasons may explain the lower complication rates reported in this analysis compared with the previous study.^3^ First, this updated analysis focused on a screening cohort, whereas the previous analysis could not make such a distinction. Second, the time window to observe complications was 3 months in this study and 12 months previously; this narrower time window may reduce the likelihood of misclassifying complications from other diseases.^4^ Third, the procedure and diagnosis codes used were revised to improve accuracy in the classification of procedure types and severity. Nevertheless, the overall complication rate from this updated analysis remained 77% higher than that reported in the NLST. This higher rate observed among a screening cohort in community settings validates the concern of higher complication rates outside the NLST.^5^ One limitation of this study was that we were not able to determine whether patients who had LDCT screening met the screening eligibility criteria because information on smoking pack-years and history was not available in claims data. As policy makers develop strategies to improve lung cancer screening rates in communities, our study highlights the importance of minimizing potential harms of screening by using shared decision-making and ensuring adherence to clinical guidelines for evaluation and management of screening-detected lung abnormalities.

## Notes

Supplement. **eTable 1.** International Classification of Diseases (ICD) 9th and 10th Revision Procedure Codes and Current Procedural Terminology (CPT) Codes Used to Determine Diagnostic Procedures **eTable 2.** International Classification of Diseases (ICD) 9th and 10th Revision and Current Procedural Terminology (CPT) Codes Used to Determine Major, Intermediate and Minor Complication Outcomes

## References

[1] Aberle DR, Adams AM, Berg CD, ; National Lung Screening Trial Research Team Reduced lung-cancer mortality with low-dose computed tomographic screening. N Engl J Med. 2011;365(5):395-409. doi:10.1056/NEJMoa1102873 21714641 PMC4356534

[2] Centers for Medicare and Medicaid Services Medicare Coverage Database. National coverage determination (NCD) for lung cancer screening with low dose computed tomography (LDCT) (210.14). Accessed November 9, 2020. https://www.cms.gov/medicare-coverage-database/details/ncd-details.aspx?NCDId=364&ncdver=1&DocID=210.14&clickon=search&bc=gAAAAAgAAAAAAA%3d%3d&

[3] Huo J, Xu Y, Sheu T, Volk RJ, Shih YT Complication rates and downstream medical costs associated with invasive diagnostic procedures for lung abnormalities in the community setting. JAMA Intern Med. 2019;179(3):324-332. doi:10.1001/jamainternmed.2018.6277 30640382 PMC6440230

[4] Halliday SJ, Aboudara MC, Maldonado F Complication rates in a study of invasive diagnostic procedures for lung abnormalities. JAMA Intern Med. 2019;179(6):846-847. doi:10.1001/jamainternmed.2019.0960 31157840

[5] Medicare Evidence Development and Coverage Advisory Committee MEDCAC meeting 4/30/2014-lung cancer screening with low dose computed tomography. Accessed November 9, 2020. https://www.cms.gov/medicare-coverage-database/details/medcac-meeting-details.aspx?MEDCACId=68

