# Supplementary material for: Updated Analysis of Complication Rates Associated With Invasive Diagnostic Procedures After Lung Cancer Screening
Source: JAMA Netw Open. 2020 Dec 16;3(12):e2029874. doi: 10.1001/jamanetworkopen.2020.29874 (PMC7745100; doi:10.1001/jamanetworkopen.2020.29874)
Supplement: Supplement. — eTable 1. International Classification of Diseases (ICD) 9th and 10th Revision Procedure Codes and Current Procedural Terminology (CPT) Codes Used to Determine Diagnostic Procedures eTable 2. International Classification of Diseases (ICD) 9th and 10th Revision and Current Procedural Terminology (CPT) Codes Used to Determine Major, Intermediate and Minor Complication Outcomes [file jamanetwopen-e2029874-s001.pdf]

SUPPLEMENTARY ONLINE CONTENT

Zhao H, Xu Y, Huo J, Burks AC, Ost DE, Shih YCT. Updated analysis of complication rates associated with invasive diagnostic procedures after lung cancer screening. *JAMA Netw Open.* 2020;3(12).e2029874. doi:10.1001/jamanetworkopen.2020.29874

eTable 1. International Classification of Diseases (ICD) 9th and 10th Revision Procedure Codes and Current Procedural Terminology (CPT) Codes Used to Determine Diagnostic Procedures

eTable 2. International Classification of Diseases (ICD) 9th and 10th Revision and Current Procedural Terminology (CPT) Codes Used to Determine Major, Intermediate and Minor Complication Outcomes

This supplementary material has been provided by the authors to give readers additional information about their work.

© 2020 Zhao H et al. *JAMA Network Open.*

eTable 1. International Classification of Diseases (ICD) 9th and 10th Revision Procedure Codes and Current Procedural Terminology (CPT) Codes Used to Determine Diagnostic Procedures

Diagnostic Procedure	ICD-9 Procedure Code	ICD-10 Procedure Code	CPT Code
**Cytology / needle biopsy procedures:**			
Cytopathology, evaluation of fine needle aspirate; immediate cytohistologic study to determine adequacy for diagnosis, first evaluation episode, each site			88172
Cytopathology, evaluation of fine needle aspirate; interpretation and report			88173
Cytopathology, evaluation of fine needle aspirate; immediate cytohistologic study to determine adequacy for diagnosis, each separate additional evaluation episode, same site (List separately in addition to code for primary procedure)			88177
Fine needle aspiration; without imaging guidance			10021
Fine needle aspiration; with imaging guidance			10022
Biopsy, pleura, percutaneous needle			32400
Biopsy, lung or mediastinum, percutaneous needle	33.26	0BB13ZX, 0BB23ZX, 0BB33ZX, 0BB43ZX, 0BB53ZX, 0BB63ZX, 0BB73ZX, 0BB83ZX, 0BB93ZX, 0BBB3ZX, 0BBC3ZX, 0BBD3ZX, 0BBF3ZX, 0BBG3ZX, 0BBH3ZX, 0BBJ3ZX, 0BBK3ZX, 0BBL3ZX, 0BBM3ZX, 0BB13ZZ, 0BB23ZZ, 0BB33ZZ, 0BB43ZZ, 0BB53ZZ, 0BB63ZZ, 0BB73ZZ, 0BB83ZZ, 0BB93ZZ, 0BBB3ZZ, 0BBC3ZZ, 0BBD3ZZ, 0BBF3ZZ, 0BBG3ZZ, 0BBH3ZZ, 0BBJ3ZZ, 0BBK3ZZ, 0BBL3ZZ, 0BBM3ZZ, 0BB14ZX, 0BB24ZX, 0BB34ZX, 0BB44ZX, 0BB54ZX, 0BB64ZX, 0BB74ZX, 0BB84ZX, 0BB94ZX, 0BBB4ZX, 0BBC4ZX, 0BBD4ZX, 0BBF4ZX, 0BBG4ZX, 0BBH4ZX, 0BBJ4ZX, 0BBK4ZX, 0BBL4ZX, 0BBM4ZX, 0BB14ZZ, 0BB24ZZ, 0BB34ZZ, 0BB44ZZ, 0BB54ZZ, 0BB64ZZ, 0BB74ZZ, 0BB84ZZ, 0BB94ZZ, 0BBB4ZZ, 0BBC4ZZ, 0BBD4ZZ, 0BBF4ZZ, 0BBG4ZZ, 0BBH4ZZ, 0BBJ4ZZ, 0BBK4ZZ, 0BBL4ZZ, 0BBM4ZZ, 0BBN3ZX, 0BBP3ZX, 0BBR3ZX, 0BBS3ZX, 0BBN3ZZ, 0BBP3ZZ, 0BBR3ZZ, 0BBS3ZZ, 0B9130Z, 0B9230Z, 0B9330Z, 0B9430Z, 0B9530Z, 0B9630Z, 0B9730Z, 0B9830Z, 0B9930Z, 0B9B30Z, 0B9C30Z, 0B9D30Z,	32405

© 2020 Zhao H et al. *JAMA Network Open.*

		0B9F30Z, 0B9G30Z, 0B9H30Z, 0B9J30Z, 0B9K30Z, 0B9L30Z, 0B9M30Z, 0B9140Z, 0B9240Z, 0B9340Z, 0B9440Z, 0B9540Z, 0B9640Z, 0B9740Z, 0B9840Z, 0B9940Z, 0B9B40Z, 0B9C40Z, 0B9D40Z, 0B9F40Z, 0B9G40Z, 0B9H40Z, 0B9J40Z, 0B9K40Z, 0B9L40Z, 0B9M40Z, 0B913ZX, 0B923ZX, 0B933ZX, 0B943ZX, 0B953ZX, 0B963ZX, 0B973ZX, 0B983ZX, 0B993ZX, 0B9B3ZX, 0B9C3ZX, 0B9D3ZX, 0B9F3ZX, 0B9G3ZX, 0B9H3ZX, 0B9J3ZX, 0B9K3ZX, 0B9L3ZX, 0B9M3ZX, 0B914ZX, 0B924ZX, 0B934ZX, 0B944ZX, 0B954ZX, 0B964ZX, 0B974ZX, 0B984ZX, 0B994ZX, 0B9B4ZX, 0B9C4ZX, 0B9D4ZX, 0B9F4ZX, 0B9G4ZX, 0B9H4ZX, 0B9J4ZX, 0B9K4ZX, 0B9L4ZX, 0B9M4ZX, 0B913ZZ, 0B923ZZ, 0B933ZZ, 0B943ZZ, 0B953ZZ, 0B963ZZ, 0B973ZZ, 0B983ZZ, 0B993ZZ, 0B9B3ZZ, 0B9C3ZZ, 0B9D3ZZ, 0B9F3ZZ, 0B9G3ZZ, 0B9H3ZZ, 0B9J3ZZ, 0B9K3ZZ, 0B9L3ZZ, 0B9M3ZZ, 0B914ZZ, 0B924ZZ, 0B934ZZ, 0B944ZZ, 0B954ZZ, 0B964ZZ, 0B974ZZ, 0B984ZZ, 0B994ZZ, 0B9B4ZZ, 0B9C4ZZ, 0B9D4ZZ, 0B9F4ZZ, 0B9G4ZZ, 0B9H4ZZ, 0B9J4ZZ, 0B9K4ZZ, 0B9L4ZZ, 0B9M4ZZ, 0B9N30Z, 0B9P30Z, 0B9R30Z, 0B9S30Z, 0B9N40Z, 0B9P40Z, 0B9R40Z, 0B9S40Z, 0B9N3ZX, 0B9P3ZX, 0B9R3ZX, 0B9S3ZX, 0B9N4ZX, 0B9P4ZX, 0B9R4ZX, 0B9S4ZX, 0B9N3ZZ, 0B9P3ZZ, 0B9R3ZZ, 0B9S3ZZ, 0B9N4ZZ, 0B9P4ZZ, 0B9R4ZZ, 0B9S4ZZ, 0W9C30Z, 0W9930Z, 0W9B30Z, 0W9C40Z, 0W9940Z, 0W9B40Z, 0W9C3ZX, 0W993ZX, 0W9B3ZX, 0W9C4ZX, 0W994ZX, 0W9B4ZX, 0W9C3ZZ, 0W993ZZ, 0W9B3ZZ, 0W9C4ZZ, 0W994ZZ, 0W9B4ZZ, 0WBC3ZX, 0WBC3ZZ, 0WBC4ZX, 0WBC4ZZ	
Closed (percutaneous) (needle) biopsy of liver	50.11	079740Z, 079840Z, 079940Z, 079D40Z, 079K40Z, 07974ZZ, 07984ZZ, 07994ZZ, 079D4ZZ, 079K4ZZ, 07974ZX, 07984ZX, 07994ZX, 079D4ZX, 079K4ZX, 07B74ZX, 07B84ZX, 07B94ZX, 07BD4ZX, 07BK4ZX, 07B74ZZ, 07B84ZZ, 07B94ZZ, 07BD4ZZ, 07BK4ZZ, 0FB03ZX, 0FB13ZX, 0FB23ZX, 0FB03ZZ, 0FB13ZZ, 0FB23ZZ, 0FB04ZX, 0FB14ZX, 0FB24ZX, 0FB04ZZ, 0FB14ZZ, 0FB24ZZ	47000
Biopsy, abdominal or retroperitoneal mass, percutaneous needle	54.24	0W9G30Z, 0W9H30Z, 0W9G40Z, 0W9H40Z, 0W9G3ZZ, 0W9H3ZZ, 0W9G4ZZ, 0W9H4ZZ, 0W9G3ZX, 0W9H3ZX, 0W9G4ZX, 0W9H4ZX, 0WBH3ZX, 0WBH3ZZ, 0WBH4ZX, 0WBH4ZZ	49180
Biopsy or excision of lymph node(s); by needle, superficial (eg, cervical, inguinal, axillary)	40.21	079130Z, 079230Z, 079530Z, 079630Z, 079730Z, 079830Z, 079930Z, 079D30Z, 079K30Z, 079M30Z, 079140Z, 079240Z, 079540Z, 079640Z, 079740Z, 079840Z, 079940Z, 079D40Z, 079K40Z, 079M40Z, 07913ZZ, 07923ZZ, 07953ZZ, 07963ZZ, 07973ZZ, 07983ZZ, 07993ZZ, 079D3ZZ, 079K3ZZ, 079M3ZZ, 07914ZZ, 07924ZZ, 07954ZZ, 07964ZZ, 07974ZZ, 07984ZZ, 07994ZZ,	38505

© 2020 Zhao H et al. *JAMA Network Open.*

		079D4ZZ, 079K4ZZ, 079M4ZZ, 07913ZX, 07923ZX, 07953ZX, 07963ZX, 07973ZX, 07983ZX, 07993ZX, 079D3ZX, 079K3ZX, 079M3ZX, 07914ZX, 07924ZX, 07954ZX, 07964ZX, 07974ZX, 07984ZX, 07994ZX, 079D4ZX, 079K4ZX, 079M4ZX, 07B13ZX, 07B23ZX, 07B53ZX, 07B63ZX, 07B73ZX, 07B83ZX, 07B93ZX, 07BD3ZX, 07BK3ZX, 07BM3ZX, 07B13ZZ, 07B23ZZ, 07B53ZZ, 07B63ZZ, 07B73ZZ, 07B83ZZ, 07B93ZZ, 07BD3ZZ, 07BK3ZZ, 07BM3ZZ, 07B14ZX, 07B24ZX, 07B54ZX, 07B64ZX, 07B74ZX, 07B84ZX, 07B94ZX, 07BD4ZX, 07BK4ZX, 07BM4ZX, 07B14ZZ, 07B24ZZ, 07B54ZZ, 07B64ZZ, 07B74ZZ, 07B84ZZ, 07B94ZZ, 07BD4ZZ, 07BK4ZZ, 07BM4ZZ	
**Bronchoscopy procedures:**			
Transbronchoscopic needle aspiration [TBNA] of lymph node	40.11	079740Z, 079840Z, 079940Z, 079D40Z, 079K40Z, 07974ZZ, 07984ZZ, 07994ZZ, 079D4ZZ, 079K4ZZ, 07974ZX, 07984ZX, 07994ZX, 079D4ZX, 079K4ZX, 07B74ZX, 07B84ZX, 07B94ZX, 07BD4ZX, 07BK4ZX, 07B74ZZ, 07B84ZZ, 07B94ZZ, 07BD4ZZ, 07BK4ZZ	
Catheterization with bronchial brush biopsy			31717
Bronchoscopy through artificial stoma (Tracheobronchoscopy through established tracheostomy incision)	33.21	0BJ07ZZ, 0BJ17ZZ, 0BJK7ZZ, 0BJL7ZZ, 0BJ08ZZ, 0BJ18ZZ, 0BJK8ZZ, 0BJL8ZZ, 0WJQ7ZZ, 0WJQ8ZZ	31615
Fiber-optic bronchoscopy	33.22	0BJ04ZZ, 0BJ14ZZ, 0BJK4ZZ, 0BJL4ZZ, 0BJ08ZZ, 0BJ18ZZ, 0BJK8ZZ, 0BJL8ZZ, 0WJQ4ZZ, 0WJQ8ZZ	
Other bronchoscopy	33.23	0BJ07ZZ, 0BJ17ZZ, 0BJK7ZZ, 0BJL7ZZ, 0BJ08ZZ, 0BJ18ZZ, 0BJK8ZZ, 0BJL8ZZ, 0WJQ7ZZ, 0WJQ8ZZ, 0BJ04ZZ, 0BJ14ZZ, 0BJK4ZZ, 0BJL4ZZ, 0WJQ4ZZ, 0WJQ8ZZ	
Closed [endoscopic] biopsy of bronchus	33.24	0B9140Z, 0B9240Z, 0B9340Z, 0B9440Z, 0B9540Z, 0B9640Z, 0B9740Z, 0B9840Z, 0B9940Z, 0B9B40Z, 0B9180Z, 0B9280Z, 0B9380Z, 0B9480Z, 0B9580Z, 0B9680Z, 0B9780Z, 0B9880Z, 0B9980Z, 0B9B80Z, 0B914ZZ, 0B924ZZ, 0B934ZZ, 0B944ZZ, 0B954ZZ, 0B964ZZ, 0B974ZZ, 0B984ZZ, 0B994ZZ, 0B9B4ZZ, 0B914ZX, 0B924ZX, 0B934ZX, 0B944ZX, 0B954ZX, 0B964ZX, 0B974ZX, 0B984ZX, 0B994ZX, 0B9B4ZX, 0B918ZZ, 0B928ZZ, 0B938ZZ, 0B948ZZ, 0B958ZZ, 0B968ZZ, 0B978ZZ, 0B988ZZ, 0B998ZZ, 0B9B8ZZ, 0B918ZX, 0B928ZX, 0B938ZX, 0B948ZX, 0B958ZX, 0B968ZX, 0B978ZX, 0B988ZX, 0B998ZX, 0B9B8ZX, 0BB14ZZ, 0BB24ZZ, 0BB34ZZ, 0BB44ZZ, 0BB54ZZ, 0BB64ZZ, 0BB74ZZ, 0BB84ZZ, 0BB94ZZ, 0BBB4ZZ, 0BB14ZX, 0BB24ZX, 0BB34ZX, 0BB44ZX, 0BB54ZX, 0BB64ZX, 0BB74ZX, 0BB84ZX, 0BB94ZX, 0BBB4ZX, 0BB18ZZ, 0BB28ZZ, 0BB38ZZ, 0BB48ZZ,	

© 2020 Zhao H et al. *JAMA Network Open.*

		0BB58ZZ, 0BB68ZZ, 0BB78ZZ, 0BB88ZZ, 0BB98ZZ, 0BBB8ZZ, 0BB18ZX, 0BB28ZX, 0BB38ZX, 0BB48ZX, 0BB58ZX, 0BB68ZX, 0BB78ZX, 0BB88ZX, 0BB98ZX, 0BBB8ZX	
Closed endoscopic biopsy of lung	33.27	0B9C40Z, 0B9D40Z, 0B9F40Z, 0B9G40Z, 0B9H40Z, 0B9J40Z, 0B9K40Z, 0B9L40Z, 0B9M40Z, 0B9C80Z, 0B9D80Z, 0B9F80Z, 0B9G80Z, 0B9H80Z, 0B9J80Z, 0B9K80Z, 0B9L80Z, 0B9M80Z, 0B9C4ZZ, 0B9D4ZZ, 0B9F4ZZ, 0B9G4ZZ, 0B9H4ZZ, 0B9J4ZZ, 0B9K4ZZ, 0B9L4ZZ, 0B9M4ZZ, 0B9C4ZX, 0B9D4ZX, 0B9F4ZX, 0B9G4ZX, 0B9H4ZX, 0B9J4ZX, 0B9K4ZX, 0B9L4ZX, 0B9M4ZX, 0B9C8ZZ, 0B9D8ZZ, 0B9F8ZZ, 0B9G8ZZ, 0B9H8ZZ, 0B9J8ZZ, 0B9K8ZZ, 0B9L8ZZ, 0B9M8ZZ, 0B9C8ZX, 0B9D8ZX, 0B9F8ZX, 0B9G8ZX, 0B9H8ZX, 0B9J8ZX, 0B9K8ZX, 0B9L8ZX, 0B9M8ZX, 0BBC4ZZ, 0BBD4ZZ, 0BBF4ZZ, 0BBG4ZZ, 0BBH4ZZ, 0BBJ4ZZ, 0BBK4ZZ, 0BBL4ZZ, 0BBM4ZZ, 0BBC4ZX, 0BBD4ZX, 0BBF4ZX, 0BBG4ZX, 0BBH4ZX, 0BBJ4ZX, 0BBK4ZX, 0BBL4ZX, 0BBM4ZX, 0BBC8ZZ, 0BBD8ZZ, 0BBF8ZZ, 0BBG8ZZ, 0BBH8ZZ, 0BBJ8ZZ, 0BBK8ZZ, 0BBL8ZZ, 0BBM8ZZ, 0BBC8ZX, 0BBD8ZX, 0BBF8ZX, 0BBG8ZX, 0BBH8ZX, 0BBJ8ZX, 0BBK8ZX, 0BBL8ZX, 0BBM8ZX	
Bronchoscopy, rigid or flexible, including fluoroscopic guidance, when performed; diagnostic, with cell washing, when performed (separate procedure)			31621, 31622, 31650
Bronchoscopy, rigid or flexible, including fluoroscopic guidance, when performed; with brushing or protected brushings			31623
Bronchoscopy, rigid or flexible, including fluoroscopic guidance, when performed; with bronchial alveolar lavage			31624
Bronchoscopy, rigid or flexible, including fluoroscopic guidance, when performed; with bronchial or endobronchial biopsy(s), single or multiple sites			31625
Bronchoscopy, rigid or flexible, including fluoroscopic guidance, when performed; with placement of fiducial markers, single or multiple			31626
Bronchoscopy, rigid or flexible, including fluoroscopic guidance, when performed; with computer-assisted, image-guided			31627

© 2020 Zhao H et al. *JAMA Network Open.*

navigation (List separately in addition to code for primary procedure[s])			
Bronchoscopy, rigid or flexible, including fluoroscopic guidance, when performed; with transbronchial lung biopsy(s), single lobe			31628
Bronchoscopy, rigid or flexible, including fluoroscopic guidance, when performed; with transbronchial needle aspiration biopsy(s), trachea, main stem and/or lobar bronchus(i)			31629
Bronchoscopy, rigid or flexible, including fluoroscopic guidance, when performed; with transbronchial lung biopsy(s), each additional lobe (List separately in addition to code for primary procedure)			31632
Bronchoscopy, rigid or flexible, including fluoroscopic guidance, when performed; with transbronchial needle aspiration biopsy(s), each additional lobe (List separately in addition to code for primary procedure)			31633
Bronchoscopy, rigid or flexible, including fluoroscopic guidance, when performed; with endobronchial ultrasound (EBUS) guided transtracheal and/or transbronchial sampling (eg, aspiration[s]/biopsy[ies]), 3 or more mediastinal and/or hilar lymph node stations or structures			31652
Bronchoscopy, rigid or flexible, including fluoroscopic guidance, when performed; with transendoscopic endobronchial ultrasound (EBUS) during bronchoscopic diagnostic or therapeutic intervention(s) for peripheral lesion(s) (List separately in addition to code for primary procedure[s])			31653
**Thoracic lung surgical procedures:**			

© 2020 Zhao H et al. *JAMA Network Open.*

Thoracoscopic excision of lesion or tissue of lung	32.20	0B910ZZ, 0B920ZZ, 0B930ZZ, 0B940ZZ, 0B950ZZ, 0B960ZZ, 0B970ZZ, 0B980ZZ, 0B990ZZ, 0B9B0ZZ, 0B9C0ZZ, 0B9D0ZZ, 0B9F0ZZ, 0B9G0ZZ, 0B9H0ZZ, 0B9J0ZZ, 0B9K0ZZ, 0B9L0ZZ, 0B9M0ZZ, 0B910ZX, 0B920ZX, 0B930ZX, 0B940ZX, 0B950ZX, 0B960ZX, 0B970ZX, 0B980ZX, 0B990ZX, 0B9B0ZX, 0B9C0ZX, 0B9D0ZX, 0B9F0ZX, 0B9G0ZX, 0B9H0ZX, 0B9J0ZX, 0B9K0ZX, 0B9L0ZX, 0B9M0ZX	
Thoracoscopy, surgical; with lobectomy (single lobe)	32.41	0BTC0ZZ, 0BTD0ZZ, 0BTF0ZZ, 0BTG0ZZ, 0BTH0ZZ, 0BTJ0ZZ, 0BTK0ZZ, 0BTL0ZZ	32663, 32670
Thoracoscopic lung biopsy	33.20	0BB10ZZ, 0BB20ZZ, 0BB30ZZ, 0BB40ZZ, 0BB50ZZ, 0BB60ZZ, 0BB70ZZ, 0BB80ZZ, 0BB90ZZ, 0BBB0ZZ, 0BBC0ZZ, 0BBD0ZZ, 0BBF0ZZ, 0BBG0ZZ, 0BBH0ZZ, 0BBJ0ZZ, 0BBK0ZZ, 0BBM0ZZ, 0BB10ZX, 0BB20ZX, 0BB30ZX, 0BB40ZX, 0BB50ZX, 0BB60ZX, 0BB70ZX, 0BB80ZX, 0BB90ZX, 0BBB0ZX, 0BBC0ZX, 0BBD0ZX, 0BBF0ZX, 0BBG0ZX, 0BBH0ZX, 0BBJ0ZX, 0BBK0ZX, 0BBM0ZX, 0BBN0ZX, 0BBP0ZX, 0BBR0ZX, 0BBS0ZX, 0BBN0ZZ, 0BBP0ZZ, 0BBR0ZZ, 0BBS0ZZ	
Thoracotomy; with exploration	34.02	0WJ80ZZ, 0WJC0ZZ, 0WJ90ZZ, 0WJB0ZZ, 0WJP0ZZ, 0BJ00ZZ, 0BJ10ZZ, 0BJK0ZZ, 0BJL0ZZ, 0BJQ0ZZ, 0BJT0ZZ	32100
Thoracoscopy, diagnostic (separate procedure); lungs, pericardial sac, mediastinal or pleural space, without biopsy	34.21	0WJ93ZZ, 0WJB3ZZ, 0WJC3ZZ, 0WJD3ZZ, 0WJQ3ZZ, 0WJ94ZZ, 0WJB4ZZ, 0WJC4ZZ, 0WJD4ZZ, 0WJQ4ZZ, 0BJ14ZZ, 0BJK4ZZ, 0BJL4ZZ, 0BJQ4ZZ, 0BJT4ZZ, 0BJ18ZZ, 0BJK8ZZ, 0BJL8ZZ, 0BJQ8ZZ, 0BJT8ZZ	32601, 32603, 32605
Mediastinoscopy	34.22	0WJC4ZZ, 0WJQ4ZZ, 0WJQ8ZZ, 0WJQ7ZZ, 0WJD3ZZ, 0WJD4ZZ	
Thoracotomy; with therapeutic wedge resection (eg, mass or nodule), each additional resection, ipsilateral (List separately in addition to code for primary procedure)			30506
Thoracotomy; with diagnostic wedge resection followed by anatomic lung resection (List separately in addition to code for primary procedure)			30507
Thoracotomy, with diagnostic biopsy(ies) of lung infiltrate(s) (eg, wedge, incisional), unilateral			32095, 32096
Thoracotomy, with diagnostic biopsy(ies) of lung nodule(s) or mass(es) (eg, wedge, incisional), unilateral			32097

© 2020 Zhao H et al. *JAMA Network Open.*

Thoracoscopy; with diagnostic biopsy(ies) of lung infiltrate(s) (eg, wedge, incisional), unilateral			32602, 32607
Thoracoscopy, diagnostic (separate procedure); pericardial sac, with biopsy			32604
Thoracoscopy, diagnostic (separate procedure); mediastinal space, with biopsy			32606, 32700, 32705
Thoracoscopy; with diagnostic biopsy(ies) of lung nodule(s) or mass(es) (eg, wedge, incisional), unilateral			32608
Thoracoscopy; with biopsy(ies) of pleura			32609
Thoracotomy, with biopsy(ies) of pleura			32402, 32098
Thoracotomy; with therapeutic wedge resection (eg, mass, nodule), initial			32500, 32505
Thoracoscopy, surgical; with therapeutic wedge resection (eg, mass, nodule), initial unilateral			32657, 32666
Thoracoscopy, surgical; with therapeutic wedge resection (eg, mass or nodule), each additional resection, ipsilateral (List separately in addition to code for primary procedure)			32667
Thoracoscopy, surgical; with diagnostic wedge resection followed by anatomic lung resection (List separately in addition to code for primary procedure)			32668
Thoracoscopy, surgical; with removal of a single lung segment (segmentectomy)	32.30	0BBC7ZZ, 0BBD7ZZ, 0BBF7ZZ, 0BBG7ZZ, 0BBH7ZZ, 0BBJ7ZZ, 0BBK7ZZ, 0BBL7ZZ, 0BBM7ZZ, 0BBC7ZX, 0BBD7ZX, 0BBF7ZX, 0BBG7ZX, 0BBH7ZX, 0BBJ7ZX, 0BBK7ZX, 0BBL7ZX, 0BBM7ZX, 0BBC8ZZ, 0BBD8ZZ, 0BBF8ZZ, 0BBG8ZZ, 0BBH8ZZ, 0BBJ8ZZ, 0BBK8ZZ, 0BBL8ZZ, 0BBM8ZZ, 0BBC8ZX, 0BBD8ZX, 0BBF8ZX, 0BBG8ZX, 0BBH8ZX, 0BBJ8ZX, 0BBK8ZX, 0BBL8ZX, 0BBM8ZX	32669
Thoracoscopy, surgical; with removal of two lobes (bilobectomy)			32670
Thoracoscopy, surgical; with mediastinal and regional lymphadenectomy (List separately in addition to code for primary procedure)			32674

© 2020 Zhao H et al. *JAMA Network Open.*

Mediastinotomy with exploration, drainage, removal of foreign body, or biopsy; cervical approach			39000
Mediastinotomy with exploration, drainage, removal of foreign body, or biopsy; transthoracic approach, including either transthoracic or median sternotomy			39010
Mediastinoscopy, includes biopsy(ies), when performed			39400
Mediastinoscopy; includes biopsy(ies) of mediastinal mass (eg, lymphoma), when performed			39401
Mediastinoscopy; with lymph node biopsy(ies) (eg, lung cancer staging)			39402
Other local excision or destruction of lesion or tissue of lung	32.29	0B510ZZ, 0B520ZZ, 0B530ZZ, 0B540ZZ, 0B550ZZ, 0B560ZZ, 0B570ZZ, 0B580ZZ, 0B590ZZ, 0B5B0ZZ, 0B5C0ZZ, 0B5D0ZZ, 0B5F0ZZ, 0B5G0ZZ, 0B5H0ZZ, 0B5J0ZZ, 0B5K0ZZ, 0B5L0ZZ, 0B5M0ZZ, 0B513ZZ, 0B523ZZ, 0B533ZZ, 0B543ZZ, 0B553ZZ, 0B563ZZ, 0B573ZZ, 0B583ZZ, 0B593ZZ, 0B5B3ZZ, 0B5C3ZZ, 0B5D3ZZ, 0B5F3ZZ, 0B5G3ZZ, 0B5H3ZZ, 0B5J3ZZ, 0B5K3ZZ, 0B5L3ZZ, 0B5M3ZZ, 0B514ZZ, 0B524ZZ, 0B534ZZ, 0B544ZZ, 0B554ZZ, 0B564ZZ, 0B574ZZ, 0B584ZZ, 0B594ZZ, 0B5B4ZZ, 0B5C4ZZ, 0B5D4ZZ, 0B5F4ZZ, 0B5G4ZZ, 0B5H4ZZ, 0B5J4ZZ, 0B5K4ZZ, 0B5L4ZZ, 0B5M4ZZ, 0B517ZZ, 0B527ZZ, 0B537ZZ, 0B547ZZ, 0B557ZZ, 0B567ZZ, 0B577ZZ, 0B587ZZ, 0B597ZZ, 0B5B7ZZ, 0B5C7ZZ, 0B5D7ZZ, 0B5F7ZZ, 0B5G7ZZ, 0B5H7ZZ, 0B5J7ZZ, 0B5K7ZZ, 0B5L7ZZ, 0B5M7ZZ, 0B518ZZ, 0B528ZZ, 0B538ZZ, 0B548ZZ, 0B558ZZ, 0B568ZZ, 0B578ZZ, 0B588ZZ, 0B598ZZ, 0B5B8ZZ, 0B5C8ZZ, 0B5D8ZZ, 0B5F8ZZ, 0B5G8ZZ, 0B5H8ZZ, 0B5J8ZZ, 0B5K8ZZ, 0B5L8ZZ, 0B5M8ZZ	
Removal of lung, other than pneumonectomy; with all remaining lung following previous removal of a portion of lung (completion pneumonectomy)	32.39	0BT30ZZ, 0BT40ZZ, 0BT50ZZ, 0BT60ZZ, 0BT70ZZ, 0BT80ZZ, 0BT90ZZ, 0BTB0ZZ, 0BTC0ZZ, 0BTD0ZZ, 0BTF0ZZ, 0BTG0ZZ, 0BTH0ZZ, 0BTJ0ZZ, 0BTK0ZZ, 0BTL0ZZ, 0BTM0ZZ, 0BT34ZZ, 0BT44ZZ, 0BT54ZZ, 0BT64ZZ, 0BT74ZZ, 0BT84ZZ, 0BT94ZZ, 0BTB4ZZ, 0BTC4ZZ, 0BTD4ZZ, 0BTF4ZZ, 0BTG4ZZ, 0BTH4ZZ, 0BTJ4ZZ, 0BTK4ZZ, 0BTL4ZZ, 0BTM4ZZ	32484, 32488
Other lobectomy of lung	32.49	0BTC0ZZ, 0BTD0ZZ, 0BTF0ZZ, 0BTG0ZZ, 0BTH0ZZ, 0BTJ0ZZ, 0BTK0ZZ, 0BTL0ZZ	

© 2020 Zhao H et al. *JAMA Network Open.*

Open biopsy of bronchus	33.25	0BB10ZZ, 0BB20ZZ, 0BB30ZZ, 0BB40ZZ, 0BB50ZZ, 0BB60ZZ, 0BB70ZZ, 0BB80ZZ, 0BB90ZZ, 0BBB0ZZ, 0BB10ZX, 0BB20ZX, 0BB30ZX, 0BB40ZX, 0BB50ZX, 0BB60ZX, 0BB70ZX, 0BB80ZX, 0BB90ZX, 0BBB0ZX, 0B9100Z, 0B9200Z, 0B9300Z, 0B9400Z, 0B9500Z, 0B9600Z, 0B9700Z, 0B9800Z, 0B9900Z, 0B9B00Z, 0B910ZZ, 0B920ZZ, 0B930ZZ, 0B940ZZ, 0B950ZZ, 0B960ZZ, 0B970ZZ, 0B980ZZ, 0B990ZZ, 0B9B0ZZ, 0B910ZX, 0B920ZX, 0B930ZX, 0B940ZX, 0B950ZX, 0B960ZX, 0B970ZX, 0B980ZX, 0B990ZX, 0B9B0ZX	
Removal of lung, other than pneumonectomy; single lobe (lobectomy)			32480
Removal of lung, other than pneumonectomy; 2 lobes (bilobectomy)			32482
Removal of lung, other than pneumonectomy; with circumferential resection of segment of bronchus followed by broncho-bronchial anastomosis (sleeve lobectomy)			32486
Resection of lung; with resection of chest wall			32520
Resection of lung; with reconstruction of chest wall, without prosthesis			32522
Resection of lung; with major reconstruction of chest wall, with prosthesis			32525
**Other surgical procedures:**			
Open biopsy of lung	33.28	0BBC0ZZ, 0BBD0ZZ, 0BBF0ZZ, 0BBG0ZZ, 0BBH0ZZ, 0BBJ0ZZ, 0BBK0ZZ, 0BBL0ZZ, 0BBM0ZZ, 0BBC0ZX, 0BBD0ZX, 0BBF0ZX, 0BBG0ZX, 0BBH0ZX, 0BBJ0ZX, 0BBK0ZX, 0BBL0ZX, 0BBM0ZX, 0B9C00Z, 0B9D00Z, 0B9F00Z, 0B9G00Z, 0B9H00Z, 0B9J00Z, 0B9K00Z, 0B9L00Z, 0B9M00Z, 0B9C0ZZ, 0B9D0ZZ, 0B9F0ZZ, 0B9G0ZZ, 0B9H0ZZ, 0B9J0ZZ, 0B9K0ZZ, 0B9L0ZZ, 0B9M0ZZ, 0B9C0ZX, 0B9D0ZX, 0B9F0ZX, 0B9G0ZX, 0B9H0ZX, 0B9J0ZX, 0B9K0ZX, 0B9L0ZX, 0B9M0ZX	
Radical dissection of thoracic structures	32.6	01B30ZZ,01BL0ZZ, 0BN10ZZ, 0BN20ZZ, 0BN30ZZ, 0BN40ZZ, 0BN50ZZ, 0BN60ZZ, 0BN70ZZ, 0BN80ZZ, 0BN90ZZ, 0BNB0ZZ, 0BNC0ZZ, 0BND0ZZ, 0BNF0ZZ, 0BNG0ZZ, 0BNH0ZZ, 0BNJ0ZZ, 0BNK0ZZ, 0BNL0ZZ, 0BNM0ZZ, 0BNN0ZZ, 0BNP0ZZ, 0BNR0ZZ, 0BNS0ZZ	

© 2020 Zhao H et al. *JAMA Network Open.*

Resection of apical lung tumor (eg, Pancoast tumor), including chest wall resection, rib(s) resection(s), neurovascular dissection, when performed; without chest wall reconstruction(s)			32503
Resection of apical lung tumor (eg, Pancoast tumor), including chest wall resection, rib(s) resection(s), neurovascular dissection, when performed; with chest wall reconstruction			32504
Biopsy or excision of lymph node(s); open, superficial	40.23	079100Z, 079200Z, 079500Z, 079600Z, 079700Z, 079800Z, 079900Z, 079D00Z, 079K00Z, 079M00Z, 079130Z, 079230Z, 079530Z, 079630Z, 079730Z, 079830Z, 079930Z, 079D30Z, 079K30Z, 079M30Z, 079140Z, 079240Z, 079540Z, 079640Z, 079740Z, 079840Z, 079940Z, 079D40Z, 079K40Z, 079M40Z, 07910ZZ, 07920ZZ, 07950ZZ, 07960ZZ, 07970ZZ, 07980ZZ, 07990ZZ, 079D0ZZ, 079K0ZZ, 079M0ZZ, 07913ZZ, 07923ZZ, 07953ZZ, 07963ZZ, 07973ZZ, 07983ZZ, 07993ZZ, 079D3ZZ, 079K3ZZ, 079M3ZZ, 07914ZZ, 07924ZZ, 07954ZZ, 07964ZZ, 07974ZZ, 07984ZZ, 07994ZZ, 079D4ZZ, 079K4ZZ, 079M4ZZ, 07910ZX, 07920ZX, 07950ZX, 07960ZX, 07970ZX, 07980ZX, 07990ZX, 079D0ZX, 079K0ZX, 079M0ZX, 07913ZX, 07923ZX, 07953ZX, 07963ZX, 07973ZX, 07983ZX, 07993ZX, 079D3ZX, 079K3ZX, 079M3ZX, 07914ZX, 07924ZX, 07954ZX, 07964ZX, 07974ZX, 07984ZX, 07994ZX, 079D4ZX, 079K4ZX, 079M4ZX, 07B10ZX, 07B20ZX, 07B50ZX, 07B60ZX, 07B70ZX, 07B80ZX, 07B90ZX, 07BD0ZX, 07BK0ZX, 07BM0ZX, 07B10ZZ, 07B20ZZ, 07B50ZZ, 07B60ZZ, 07B70ZZ, 07B80ZZ, 07B90ZZ, 07BD0ZZ, 07BK0ZZ, 07BM0ZZ, 07B13ZX, 07B23ZX, 07B53ZX, 07B63ZX, 07B73ZX, 07B83ZX, 07B93ZX, 07BD3ZX, 07BK3ZX, 07BM3ZX, 07B13ZZ, 07B23ZZ, 07B53ZZ, 07B63ZZ, 07B73ZZ, 07B83ZZ, 07B93ZZ, 07BD3ZZ, 07BK3ZZ, 07BM3ZZ, 07B14ZX, 07B24ZX, 07B54ZX, 07B64ZX, 07B74ZX, 07B84ZX, 07B94ZX, 07BD4ZX, 07BK4ZX, 07BM4ZX, 07B14ZZ, 07B24ZZ, 07B54ZZ, 07B64ZZ, 07B74ZZ, 07B84ZZ, 07B94ZZ, 07BD4ZZ, 07BK4ZZ, 07BM4ZZ	38500
Biopsy or excision of lymph node(s); open, deep cervical node(s)			38510

© 2020 Zhao H et al. *JAMA Network Open.*

Biopsy or excision of lymph node(s); open, deep cervical node(s) with excision scalene fat pad			38520
Biopsy or excision of lymph node(s); open, deep axillary node(s)			38525
Radical excision of cervical lymph nodes	40.40, 40.41, 40.42	07T10ZZ, 07T14ZZ, 07T20ZZ, 07T24ZZ	38720, 38724
Axillary lymphadenectomy; superficial	40.51	07T50ZZ, 07T54ZZ, 07T60ZZ, 07T64ZZ	38740
Axillary lymphadenectomy; complete			38745

Abbreviations: CPT (current procedural terminology), ICD (international classification of diseases)

© 2020 Zhao H et al. *JAMA Network Open.*

eTable 2. International Classification of Diseases (ICD) 9th and 10th Revision and Current Procedural Terminology (CPT) Codes Used to Determine Major, Intermediate and Minor Complication Outcomes

Complication	ICD-9 Diagnosis Code	ICD9 Procedure Code	ICD-10 Diagnosis Code	ICD-10 Procedure Code	CPT Code
**Major complications:**					
Acute respiratory failure/arrest<br>- following trauma and surgery<br>- acute/chronic post procedural<br>- intraoperative<br>- requiring reintubation<br>- with hypoxemia/hypoxia<br>- with hypercapnea<br>- acute on chronic<br>- acute/chronic w/ hypoxia<br>- acute/chronic w/ hypercapnia	518.81, 518.51, 518.84, 518.53, 799.1	96.04, 96.7	J96.90, R09.2, J95.821, J95.822, J95.88, J96.00, 96.01, J96.02, J96.20, J96.21, J96.22	0BH17EZ, 0BH18EZ	31500, 94660, 94644, 94645
Anaphylaxis<br>-to latex	995.0		T78.2XXA, T65.811A		
Bronchopulmonary fistula<br>- postprocedure pulm air leak<br>- persistent air leak<br>- PTX with air leak	510, 510.0	34.73	J95.812, J93.82, J93	0BQ30ZZ, 0BQ33ZZ, 0BQ34ZZ, 0BQ37ZZ, 0BQ38ZZ, 0BQ40ZZ, 0BQ43ZZ, 0BQ44ZZ, 0BQ47ZZ, 0BQ48ZZ, 0BQ50ZZ, 0BQ53ZZ, 0BQ54ZZ, 0BQ57ZZ, 0BQ58ZZ, 0BQ60ZZ, 0BQ63ZZ, 0BQ64ZZ, 0BQ67ZZ, 0BQ68ZZ, 0BQ70ZZ, 0BQ73ZZ, 0BQ74ZZ, 0BQ77ZZ, 0BQ78ZZ, 0BQ80ZZ, 0BQ83ZZ, 0BQ84ZZ, 0BQ87ZZ, 0BQ88ZZ, 0BQ90ZZ, 0BQ93ZZ, 0BQ94ZZ, 0BQ97ZZ, 0BQ98ZZ, 0BQB0ZZ, 0BQB3ZZ, 0BQB4ZZ, 0BQB7ZZ, 0BQB8ZZ, 0WQC0ZZ,	32815

© 2020 Zhao H et al. *JAMA Network Open.*

				0BQN0ZZ, 0BQN3ZZ, 0BQN4ZZ, 0BQP0ZZ, 0BQP3ZZ, 0BQP4ZZ	
Cardiac arrest<br>- following intubation<br>- due to respiratory disorder<br>- due to cardiac disorder	997.1	99.60	I46, I46.9, I97.89, I97.710, J98.9, I46.8 I46.2	5A12012	92950
Cerebral vascular accident (CVA)/stroke<br>-hemorrhagic	433-436, 438.XX, 433.XX	38.01	I63.9<br>I61.9	03CG0Z6, 03CG0ZZ, 05CL0ZZ, 05CL4ZZ	35301, 35501, 35506 - 35508, 37195
Congestive heart failure (CHF)<br>- acute LV diastolic<br>- acute LV systolic<br>- acute RV	428.x		I50.9, I50.82<br>I50.31, I50.1<br>I50.21, I50.23<br>I50.811, I50.813, I50.814		
Hemothorax<br>- postoperative	998.11	34.04, 34.06	J94.2, J95.61, J95.62, J95.830, J95.831<br>J95.89, J94.2	0W9930Z, 0W9B30Z, 0W9940Z, 0W994ZZ<br>0W9B40Z, 0W9B4ZZ, 0B9N0ZZ, 0B9N80Z,	32556, 32557, 32554, 32555, 32550, 32551
Myocardial infarction<br>-Type 1 (ST-elevation/STEMI)<br>- Type 2 (demand, non-STEMI)	410.x		I21, I21.9, I21.0, I21.1, I21.01, I21.02<br>I21.2, I21.3, I21.11, I21.29, I21.21, I21.19, I21.09<br>I21.A1, I21.4		33510, 33511, 33512, 33513, 33514, 33516, 37236, 37237, 92933, 92934, 92928, 92929
Respiratory arrest	799.1	96.04, 96.05	R09.2	0BH07DZ, 0BH07YZ, 0BH18YZ, 0BH17YZ, 0BH17EZ, 0BH18EZ	31500, 94002, 94656, 94003
Wound dehiscence<br>- postoperative	998.31, 998.32		T81.30XA, T81.32XA T81.31XA		
Empyema<br>-with fistula	510.0, 510.9	34.09	J86.9, J86.0	0B9N80Z, 0B9N0ZZ, 0B9P80Z, 0B9P0ZZ, 0BHQ0YZ, 0BHQ3YZ, 0W9900Z, 0W990ZZ, 0W9B00Z, 0W9B0ZZ	32540, 32035, 32036
Injury to vital organ or vessel<br>- injury to vessel/organ<br>- injury to thoracic vessel	861, 861.1X, 861.3X, 862, 862.1X, 862.3X, 862.8, 862.9		T14.8XXA, S25.90XA, S45.902A, S36.90XA S27.9XXA		

© 2020 Zhao H et al. *JAMA Network Open.*

- injury to pulmonary vessel<br>- injury abdominal organ<br>- injury thoracic organ					
Prolonged mechanical ventilation<br>- failure to wean<br>- inability to maintain spont. vent		96.71, 96.72	Z99.11, R06.89	5A1935Z, 5A1945Z, 5A1955Z	94002, 94003
Thromboembolic complications requiring intervention<br>- PE with cor pulmonale<br>- venous thromboembolism	415.1, 415.11, 415.12, 415.13, 415.19		I26.99, I26.09, I82.90		37184, 37185, 37186, 37187, 37188, 37191, 37192, 37193
Chylous fistula<br>- chylous effusion<br>- fistula of lymph node/vessel/thoracic duct	457.8	40.61 - 40.64	J94.0, I89.8	079K00Z, 079K40Z, 079K0ZZ, 079K4ZZ, 07QK0ZZ, 07QK3ZZ, 07QK4ZZ, 07LK0CZ, 07LK0DZ, 07LK0ZZ, 07LK3CZ, 07LK3DZ, 07LK3ZZ, 07LK4CZ, 07LK4DZ, 07LK4ZZ	38380, 38381, 38382
Brachial plexopathy	353.0		G54.0		
Lung collapse<br>- both lungs	518.0	34.06	J98.19, J98.11	0W9940Z, 0W994ZZ, 0W9B40Z, 0W9B4ZZ	32551
Infarcted sigmoid colon<br>- sigmoid colon injury	557.0	17.36, 45.76	K55.049, S36.503A	0DTN4ZZ, 0DTN0ZZ, 0DTN7ZZ, 0DTN8ZZ, 0DTNFZZ	44204, 44206, 44145, 44143, 44146
**Intermediate complications:**					
Blood loss requiring a transfusion<br>- postoperative hemorrhage<br>- pulmonary hemorrhage	287.4, V58.2, 998.1, 998.11, 285.1	99.0, 99.03, 99.04	R58, I97.620, R04.89 R95.61, R95.62, R95.830, R95.831	30233H1, 30243H1, 30253H1, 30263H1, 30233P1, 30243N1, 30243P1, 30253N1, 30253P1, 30263N1, 30263P1	36430

© 2020 Zhao H et al. *JAMA Network Open.*

- postop resp. hemorrhage					
Cardiac arrhythmia requiring medical attention<br>- during surgery	427.X	99.62	I49.9, I97.790	5A2204Z	92953, 92960, 92961
Fever requiring antibiotics<br>- post-procedure fever<br>- fever NOS	780.62		R50.82, R50.9		
Pneumothorax requiring tube placement<br>- acute spontaneous<br>- NOS<br>- iatrogenic PTX	512, 512.1, 512.2, 512.89	34.04, 34.06	J95.88, J93.83, J95.811, J93.9	0W9940Z, 0W994ZZ, 0W9B40Z, 0W9B4ZZ	32551, 32960
Rib fracture(s) – single<br>- multiple	807.XX		S22.39XA, S22.49XA		
Infections requiring antibiotics<br>- Wound infection post op<br>- UTI<br>- Sepsis NOS	599.0, 590.1, 595.3, 997.39, 997.32, 997.31, 995.91, 999.39, 995.92		T81.49XA, N39.0, N30.30, N30.31, A41.9, R65.2		
Cardiac ischemia/ST elevation	411.89, 411.81		I24.8, I24.0		
Bronchitis<br>- Acute bronchitis	490, 490.0, 466, 466.0, 507		J40, J20.9		
Pneumonia<br>-vent assoc. pneumonia (VAP)	486, 997.39, 997.32		J95.851		
Pleural effusion<br>- other pleural conditions<br>- pleural effusion in other cond.	511.1, 511.8, 511.89, 511.9	34.91, 34.04, 34.06	J90, J94.8, J91.8	0B9N8ZZ, 0B9P8ZZ, 0W993ZZ, 0W9B3ZZ, 0W9930Z, 0W9B30Z, 0W9940Z, 0W994ZZ, 0W9B40Z, 0W9B4ZZ	32556, 32557, 32554, 32555, 32550, 32551

© 2020 Zhao H et al. *JAMA Network Open.*

Severe Sepsis	995.92		R65.2		
Respiratory distress<br>- dyspnea<br>- ARDS (acute resp. dist. Synd)<br>- post-procedure pulm insuff	786.09, 518.82, 518.52		R06.03, R06.00, R06.09, J80, J95.1, J95.2, J95.3		
Splenomegaly with splenic infarcts	289.59		D73.5		
Mucous plug requiring bronchoscopy	934.0		T17.400A, T17.408, T17.410A, T17.418, T17.420, T17.428, T17.490A, T17.498A		
Steroid-induced diabetes	249, 249.0, 249.0		E09.9		
**Minor complications:**					
Allergic reaction	995.3, 995.27		T78.40XA, T50.995A		
Acute Bronchospasm	519.11		J98.01		
Vasovagal reaction<br>-hypotension	780.2, 458.9, 796.3, 458.29, 458.8		R40.4, I95.9, R03.1, I95.89		
Subcutaneous emphysema	958.7, 998.81		T97.7XXA, T81.82XA		
Atelectasis	518.0, 518,		J98.11		
Pneumothorax with no chest tube	512, 512.0, 512.8				
Ileus<br>- obstruction of duodenum	560.1, 537.2		K56.0, K56.7, K35.5		
Seroma	998.13		J95.862, J95.863, L76.34, M96.843		
Paresthesias/hyperesthesias	782.0		R20.1, R20.2, R20.3, R20.8, R20.9		

Abbreviations: CPT (current procedural terminology), ICD (international classification of diseases).

© 2020 Zhao H et al. *JAMA Network Open.*
